# Of Rodents and Primates: Time-Variant Gain in Drift–Diffusion Decision Models

**DOI:** 10.1007/s42113-023-00194-1

**Published:** 2024-01-11

**Authors:** Abdoreza Asadpour, Hui Tan, Brendan Lenfesty, KongFatt Wong-Lin

**Affiliations:** 1 Intelligent Systems Research Centre, School of Computing, Engineering and Intelligent Systems, Ulster University, Magee Campus, Derry~Londonderry, Northern Ireland UK; 2 Département Electronique et Technologies Numériques, Polytech Nantes, Nantes Université, Nantes, France

**Keywords:** Perceptual decision-making, Time-variant gain, Urgency signal, Cognitive computational modelling, Drift–diffusion model

## Abstract

Sequential sampling models of decision-making involve evidence accumulation over time and have been successful in capturing choice behaviour. A popular model is the drift–diffusion model (DDM). To capture the finer aspects of choice reaction times (RTs), time-variant gain features representing urgency signals have been implemented in DDM that can exhibit slower error RTs than correct RTs. However, time-variant gain is often implemented on both DDM’s signal and noise features, with the assumption that increasing gain on the drift rate (due to urgency) is similar to DDM with collapsing decision bounds. Hence, it is unclear whether gain effects on just the signal or noise feature can lead to a different choice behaviour. This work presents an alternative DDM variant, focusing on the implications of time-variant gain mechanisms, constrained by model parsimony. Specifically, using computational modelling of choice behaviour of rats, monkeys, and humans, we systematically showed that time-variant gain only on the DDM’s noise was sufficient to produce slower error RTs, as in monkeys, while time-variant gain only on drift rate leads to faster error RTs, as in rodents. We also found minimal effects of time-variant gain in humans. By highlighting these patterns, this study underscores the utility of group-level modelling in capturing general trends and effects consistent across species. Thus, time-variant gain on DDM’s different components can lead to different choice behaviours, shed light on the underlying time-variant gain mechanisms for different species, and can be used for systematic data fitting.

**Supplementary Information:**

The online version contains supplementary material available at 10.1007/s42113-023-00194-1.

## Introduction

Choice accuracy and response times, especially in perceptual decision-making, have often been mathematically modelled by the drift–diffusion process (Ratcliff et al., 2016). There is neural evidence (Roitman & Shadlen, 2002) and underlying computational principles (e.g. Bogacz et al. (2006) and Gold and Shadlen (2007)) that support noisy temporal accumulation of evidence over time during decision formation. In this context, the drift–diffusion model (DDM) has emerged as a popular framework for describing decision-making dynamics (Ratcliff et al., 2016). In reaction time (RT) tasks, in which participants report their choices freely, the DDM is commonly used to account for the observed choice accuracy (psychometrics) and RTs (chronometrics). While the standard DDM has been extensively applied and studied, there is an ongoing need for alternative models that can capture more nuanced aspects of decision-making behaviour.

The standard DDM can be described by a stochastic differential equation (Wiener process) (Ratcliff, 1978; Ratcliff et al., 2016):

1 $$dX=\mu dt+ \sqrt{dt} \sigma \eta$$

where $X$ denotes some internal decision variable, $\mu$ is some drift rate (velocity), $\eta$ is a random variable that follows a Gaussian distribution with a mean of 0 and standard deviation of 1, $\sigma$ is the noise size (standard deviation), and $t$ is the time with time step $dt$. External stimulus or signal is encoded in the drift rate $\mu ,$ while the noise term ($\sigma \eta$) can be due to external stimulus (Ratcliff et al., 2016) or noise within the brain (Faisal et al., 2008), or both (Wang, 2002).

It should be noted in Eq. (1) the additive influence of signal and noise on the integrative process. During decision formation in a two-choice task, the decision variable $X$ has to be integrated over time in Eq. (1) such that it reaches either a prescribed upper or lower decision bound or threshold, symbolising one of the two choices being made (assuming that the initial decision variable $X$ lies between these two bounds or thresholds). It is immediately clear that error choices in the model arise purely from the noise term. Moreover, the simplicity of the model is conducive to elegant closed-form analytical solution derivations or approximations (e.g. Broderick et al., 2009; Murphy et al., 2016; Ratcliff, 1978; Smith & Ratcliff, 2022).

Averaging across trials, the standard DDM is known to produce equal mean correct and error RTs for the same signal-to-noise ratio (e.g. for the same task difficulty) (Ratcliff, 1978). Long-tailed RT distributions are also typical. Hence, standard DDM cannot account for experimental data with slower or faster error RTs or shorter tail RT distributions (Ratcliff et al., 2016). These could potentially be caused by some sense of urgency, i.e. urge to make a choice (e.g. forced to report a choice in the presence of a set time deadline) (Harris & Hutcherson, 2022). Thus, additional features are required to be incorporated into the basic DDM, leading to various DDM variants.

An approach to account for faster error RTs than correct RTs is to vary the starting point of the DDM across trials (Nguyen & Reinagel, 2022; Ratcliff & Rouder, 1998), akin to some prior noisy initial bias. To enable slower error RTs than correct RTs, one route is to have the DDM’s drift rate vary across trials such that the overall error RTs are slower than that of correct RTs (Nguyen & Reinagel, 2022; Ratcliff & Rouder, 1998). Alternatively, by decreasing the DDM’s decision bound or threshold over time (collapsing bound or threshold) within a trial, slower error than correct RTs and shorter tail RT distributions can be produced—this mimics a form of urgency signal (Ditterich, 2006; Drugowitsch et al., 2012; Hawkins et al., 2015).

Another intuitively equivalent approach is to increase the drift rate over time via a time-variant gain modulation mechanism (Ditterich, 2006; Smith & Ratcliff, 2009; Standage et al., 2011; Zhou et al., 2009). In such models, the variance grows faster than the drift rate such that later choices are more inaccurate. Mathematically, the urgency signal can be implemented either as a time-variant multiplicative factor on the signal and noise (e.g. Ditterich, 2006) or as an additive time-variant input signal (e.g. Kelly et al., 2021; Murphy et al., 2016). Multiplicative time-variant gain modulation can also be considered a form of gating, attentional, or arousal enhanced signal (e.g. Cisek et al., 2009; Niyogi & Wong-Lin, 2013; Smith & Ratcliff, 2009; Thura et al., 2012).

**Fig. 1:**
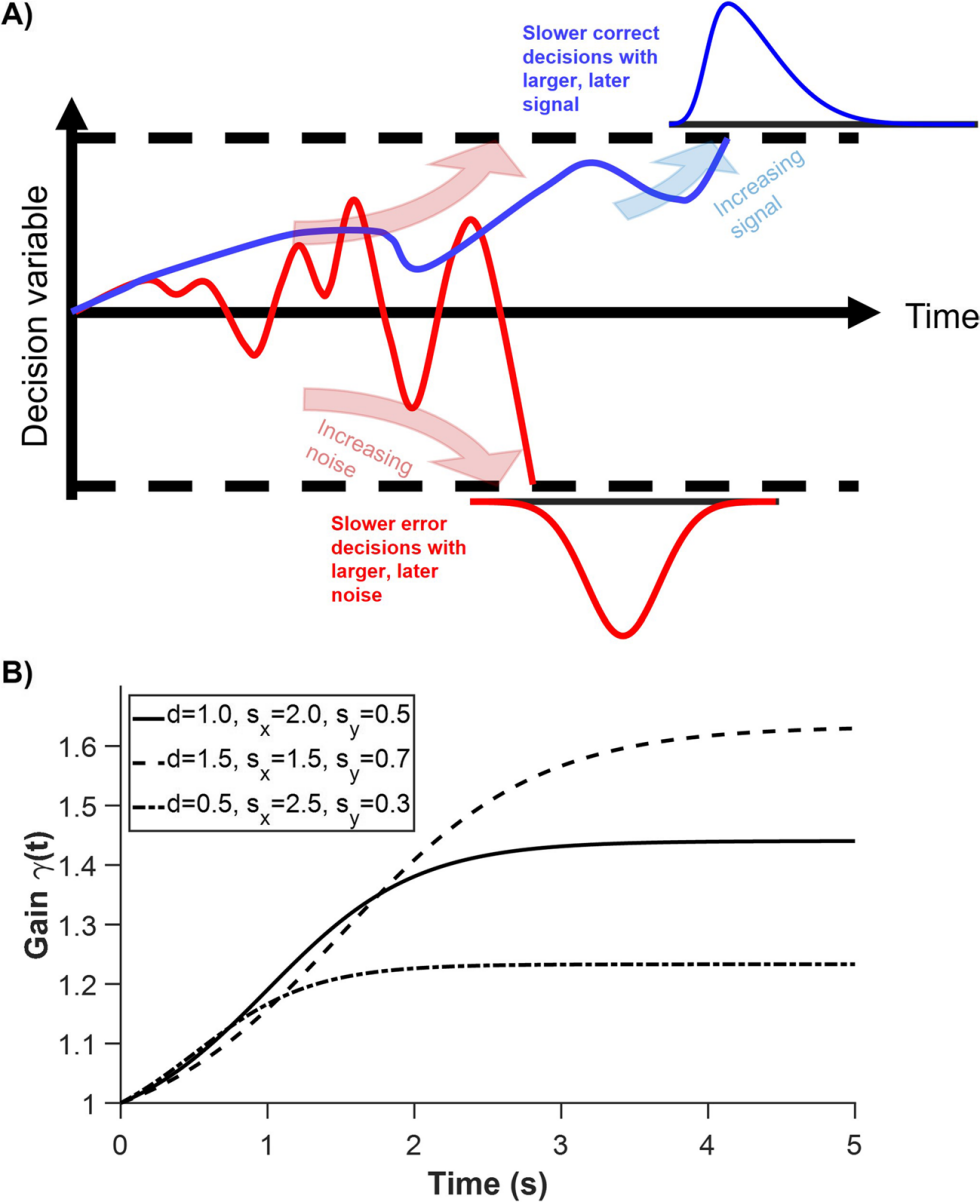
Schematic diagram to illustrate the different effects on DDM’s choice behaviour with time-variant gain on drift rate or noise. **A** Blue: increasing only signal strength over time leads to more correct decisions at a later time—slower correct choices than error choices. Red: increasing only noise level over time causes errors at a later time to be more likely—slower error choices than correct choices. Dashed lines: decision thresholds in which a choice is committed upon reaching one of them. **B** Time-variant gain function $\gamma (t)$ evolving over time for different parameter sets

In many instances, time-variant gain modulation is implemented on both the drift rate and noise term of a DDM (e.g. Ditterich, 2006). Therefore, it is unclear what the behavioural consequences would be if time-variant gain exclusively affects the DDM’s drift rate or noise. Based on a fitted time-dependent gain DDM (model 4) in Ditterich (2006), we have previously observed, using computational simulations, that time-variant gain only in the noise term of this DDM leads to slower error RTs, while time-variant gain only in the drift rate (i.e. signal) of the DDM leads to faster error RTs (Tan et al., 2023) (illustrated in Fig. 1A). However, as this was based on modifications of a model in Ditterich (2006) with parameters originally fitted to monkey data (Roitman & Shadlen, 2002), further evidence is needed to validate those results.

To address this, in this study, we aimed to contribute to introducing an alternative parsimonious model with the ability of capturing general trends and effects of decision-making behaviour consistent across subjects in different species. We used different time-variant gain DDMs, optimally and separately fitted to choice behavioural data of three species (rats, monkeys, and humans) performing the same visual motion direction discrimination task. In particular, we demonstrated that, indeed, time-variant gain just on the drift rate could account for rat data with faster error RTs, but applying this gain only to the noise term might be suitable to account for slower error RTs (as in monkey data). For human data, time-variant gain might have minimal effects.

## Methods

### Data Description

This study utilised three separate datasets from three different species. All three datasets involved the standard random-dot kinematogram in which the species had to visually discriminate the direction of coherent motion of stochastic moving dots. Task difficulty was controlled such that with lower percentage of dots moving coherently (i.e. lower motion strength) in the same direction, the task became more difficult. Both choice accuracy and RT were recorded for each motion strength (pseudo-randomly presented across trials).

The first dataset was obtained from the classic study by Roitman and Shadlen (2002), in which two macaque monkeys were used. Although both single neuronal activities were recorded, we only considered the choice behavioural data. This was also the dataset used by Ditterich (2006). The second and third datasets were from Shevinsky and Reinagel (2019) in which 51 humans and 11 rats performed the same motion discrimination task as in Roitman and Shadlen (2002). For further details, refer to the original papers.

### Description of Models

The parameter values of the one-dimensional DDM used were directly based on a model in Ditterich (2006), which readily accounted for the behaviour and (selected time sections of the) neuronal activity time course of non-human primates in a classic two-alternative, forced-choice RT task (Roitman & Shadlen, 2002). In our investigation, we aim to extend this model by focusing on time-variant gain mechanisms as an alternative modelling approach to explore the implications of time-variant gain on decision-making while maintaining model parsimony. In particular, we only focused on model 4 in Ditterich (2006), in which the decision bounds or thresholds are constant and time-variant gain modulation multiplied both the drift rate and noise term of the DDM.

In the presence of an urgency signal, $\gamma (t)$, the model is modified such that

2 $$dX=\left({\mu }_{{\mathrm{initial}}} \gamma \left(t\right)\right) dt+ \sqrt{dt} \left(\gamma \left(t\right) {\sigma }_{{\mathrm{initial}}} \right) \eta$$

where ${\mu }_{{\mathrm{initial}}}=k c$, and $c$ is the motion strength (coherence) ($c=1$ for fully coherent motion), while $k$ is some proportional constant. The noise level (standard deviation), ${\sigma }_{{\mathrm{initial}}}={\sigma }_{0} \sqrt{1+\left|c\right|}$, for some constant ${\sigma }_{0}$. The time-variant gain function was described by a logistic function, constrained to be 1 at time 0, but with an additional parameter allowing scaling of the gain range (Ditterich, 2006):

3 $$\gamma \left(t\right)=\frac{{s}_{{\mathrm{y}}}\mathrm{ exp}({s}_{{\mathrm{x}}} (t-d))}{1+{\mathrm{exp}}({s}_{{\mathrm{x}}} (t-d))}+\frac{1+\left(1-{s}_{{\mathrm{y}}}\right)\mathrm{ exp}(-{s}_{{\mathrm{x}}} d)}{1+{\mathrm{exp}}\left(-{s}_{{\mathrm{x}}} d\right)}$$

where $d$, ${s}_{{\mathrm{x}}}$, and ${s}_{{\mathrm{y}}}$ are model parameters. Figure 1B illustrates how the parameters affect the shape of this function.

Note that the variance was assumed to increase with the absolute value of the motion strength $c$, with twice the variance for a fully coherent stimulus as compared to a pure noise stimulus. If this dependency is removed, the model could still readily fit the experimental data of Roitman and Shadlen (2002), albeit not as well (Ditterich, 2006). Hence, for clarity of argument, we later removed from the noise term this dependency of $c$ such that the noise term only depended on ${\sigma }_{0}$, as follows:

4 $$dX=\left({\mu }_{{\mathrm{initial}}} \gamma \left(t\right)\right) dt+ \sqrt{dt} \left(\gamma \left(t\right) {\sigma }_{0}\right) \eta$$

The decision formation process for the standard DDM starts with an initial value of 0. The upper threshold for a correct choice (for positive drift rates) is when $X$ reaches some decision threshold, $Z=1$, while the lower threshold for an error choice (for positive drift rates) is when $X$ reaches $Z=-1$. Once a threshold is reached, the integration process is ceased, and the time duration from stimulus onset is defined as the decision time, ${t}_{{\mathrm{decision}}}$. The response time $RT$, which has been measured by Roitman and Shadlen (2002), is given by $RT={t}_{{\mathrm{decision}}}+{t}_{{\mathrm{residual}}}$. In Ditterich (2006), ${t}_{{\mathrm{residual}}}$ is assumed to be normally distributed with mean ${\overline{t} }_{{\mathrm{residual}}}$ and standard deviation ${\sigma }_{{\mathrm{residual}}}$.

Based on this model, we explored the following four cases: (i) time-variant gain on both drift rate and noise term with noise depending on $c$ (to act as the control condition); (ii) time-variant gain on both drift rate and noise term with noise independent of $c$; (iii) time-variant gain only on noise term (independent of $c$); and (iv) time-variant gain only on drift rate (with noise term independent of $c$).

### Model Fitting and Statistical Analyses

For model fitting, we opted for group-level analysis. This approach was chosen to effectively capture general trends and effects consistent across participants within each species. By focusing on group-level patterns, we aim to identify broad behavioural trends in decision-making that are common across rats, monkeys, and humans.

To feed real data into PyDDM (Shinn et al., 2020) for model 4 in Ditterich (2006), we converted RTs and other time-related parameters from milliseconds to seconds. Then, utilising the monkey dataset from Roitman and Shadlen (2002), we selected epochs with RTs between 0.1 and 1.65 s, as considered in Huk and Shadlen (2005). For the human and rat datasets from Shevinsky and Reinagel (2019), we chose the best unbiased epochs with RTs between 0.1 and 2.5 s.

In our analyses, we employed the ‘LossRobustLikelihood’ function from the PyDDM package for model fitting. This function calculates the negative log likelihood of the probability distribution functions (PDFs) in the data, thereby incorporating robustness to outliers. Contrary to approaches that focus solely on mean RT and accuracy, this likelihood-based approach assesses the probability of the entire observed dataset given the parameters of the model. This methodology ensures that our fitting procedure accounts for the entire distribution of RTs and choices, rather than just their summary statistics.

For each dataset, we fitted all four cases of the time-variant gain DDM for a trial duration of 5 s and $dt=0.01$ s using the PyDDM solver, a flexible and user-friendly software for simulating and fitting generalised DDMs (Shinn et al., 2020). Due to the stochastic nature of the PyDDM solver and the possibility of different estimated parameters for each run, we executed the code 18 times using a robust negative log likelihood as the loss function. For the next step, we generated a model squared error distribution for each case. Using the Kruskal–Wallis test (Kruskal & Wallis, 1952) as well as the pairwise comparisons with Tukey’s honestly significant difference (HSD) procedure (Tukey, 1949) and assuming the same squared error distribution for all cases, we then determined if there were statistically significant differences in the cases’ squared errors. The case with the lowest mean squared error over all runs was considered the best-fitted case. For further analysis in each case, we selected the fitted model with the smallest squared error among the runs. Since we have seven parameters for all cases, to investigate the models’ complexity cost, we calculated Akaike information criterion (AIC) (Akaike, 1974) for each case using the following equation:

5 $$AIC=14-2\times(\log\;\mathrm{likelihood})$$

For RT PDFs, motion strengths of 3.2% and 12.8% for monkeys, 2% and 12% for humans, and 10% and 50% for rats were selected to represent the relatively difficult and easy tasks, respectively, with a sufficient number of trials to generate smooth RT distributions.

### Hardware, Software, and Codes and Data Accessibility

For model fitting, we used Python 3, PyCharm 2023.1.2, and PyDDM 0.7.0, whereas for statistical analyses and plots, we used MATLAB version 2023a. Windows machines with 14 CPU cores, Intel i9-13900H, and 64-GB RAM were used.

The source codes, generated data, and analyses that support the findings of this study are available at https://github.com/abasadpour/UrgencyDDMonSpecies. The PyDDM solver is available via Shinn et al. (2020). The original datasets of the monkeys, humans, and rats are openly available via the original studies (Roitman & Shadlen, 2002; Shevinsky & Reinagel, 2019).

## Results

Our investigative approach focused on identifying the optimal model parameters for time-variant gain DDM on the choice behavioural data of three different species. Specifically, we explored the following four types or cases of the model (model 4, in Ditterich (2006)). Case (i) had time-variant gain on both drift rate and noise term with noise depending on the motion coherence, $c$, of the stimulus, as in Ditterich (2006). This would act as the control condition. Case (ii) was a slight variation of Case (i), with time-variant gain on both drift rate and noise term, but with the noise independent of $c$. The latter eliminated a possible confounding factor, while branching off into two other cases: Case (iii) had time-variant gain only on noise term (independent of $c$), while Case (iv) had time-variant gain only on drift rate with noise term independent of $c$.

Next, we used the PyDDM optimisation algorithm (Shinn et al., 2020) to fit all of the above four cases separately to each behavioural dataset per species. In particular, we used the monkey data from Roitman and Shadlen (2002) and the human and rat data from Shevinsky and Reinagel (2019). Importantly, all three datasets made use of the same experimental task paradigm, a visual motion discrimination task using standard random-dot kinematogram stimulus in a reaction time setting. We shall discuss the model fitting results in the following species order: monkeys, humans, and then rats.

### Time-Variant Gain on Both Drift Rate and Noise Accounts for Monkey Data Best

**Fig. 2:**
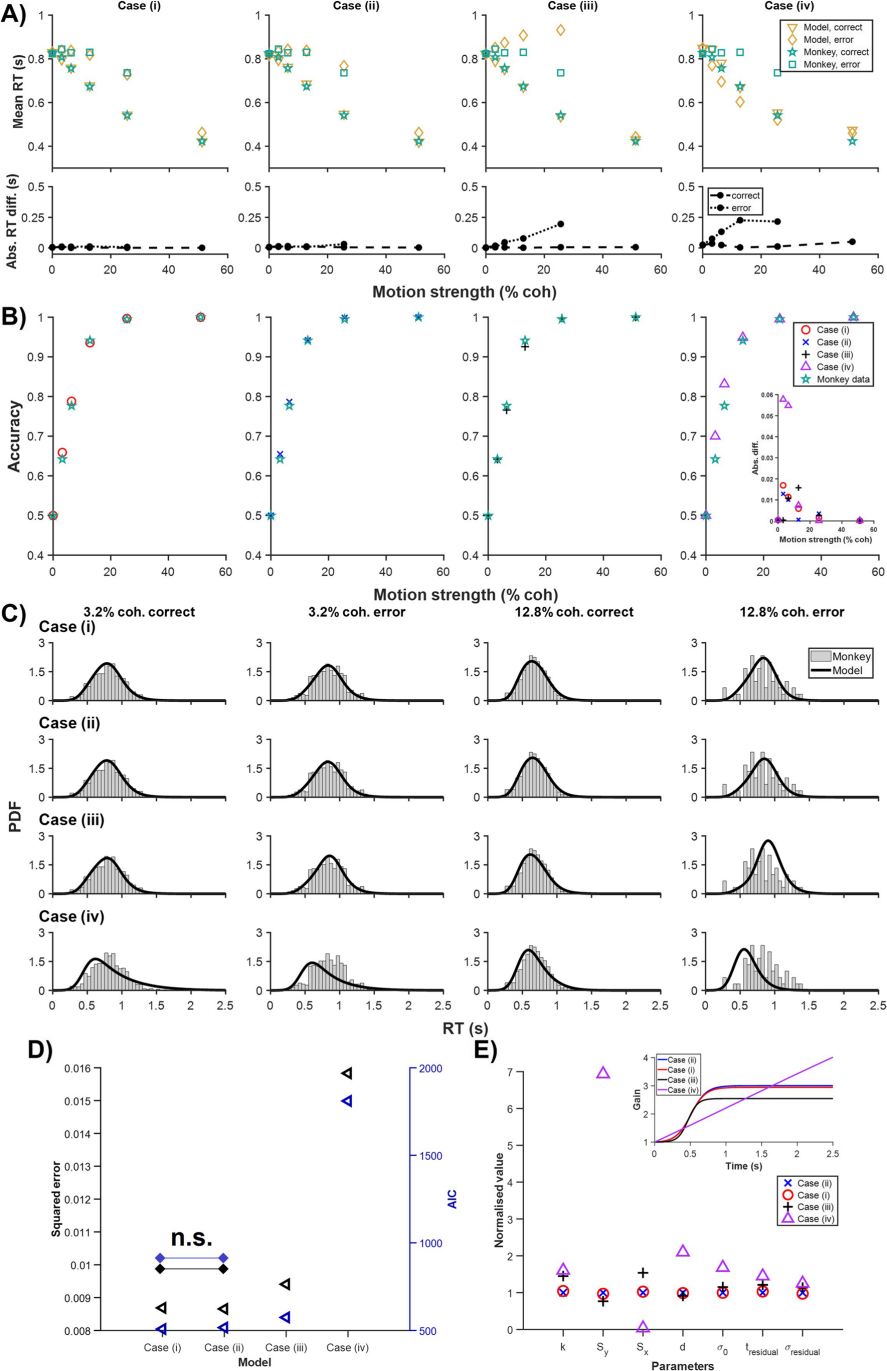
Time-variant gain on both drift rate and noise fits monkey data best. Estimated models for four cases based on monkey choice behavioural data (choice accuracy and reaction time (RT) distributions) in Roitman and Shadlen (2002). **A** Insets: absolute differences between the fitted and actual mean RTs. **B** Cases in the same order as in **A**. Inset: absolute differences between the fitted and actual choice accuracies. **C** Probability density functions (PDFs) of the correct and error RTs for selected motion strengths. **D** Model squared error and model AIC for each case. **E** Normalised values of the model parameters for the four cases. Legend order based on ranking of model fitting in **D**, best at the top. Inset: timecourse of fitted time-variant gain function for each case. Time from stimulus onset

Figure 2A shows the trend of the mean correct and error RTs across different motion strengths of the monkey data and all four cases of the model. By visual inspection, one could observe that Cases (i) and (ii) provided the best RT fit, with slower error than correct RTs. Case (iii) also had slower error RTs, albeit poorer fit. In particular, for intermediate-motion strengths, the model had much higher error RTs compared to monkey data. Case (iv) gave the worst fit, with faster error RTs. These were also indicated in the insets in Fig. 2A, which show the absolute differences between the fitted and actual mean RTs.

In terms of choice accuracy, Cases (i)–(iii) readily fitted the data, but Case (iv) performed more poorly for lower motion strengths (Fig. 2B). The inset in Fig. 2B clearly indicated this. By comparing the RT probability density functions (directly related to RT histograms) (Fig. 2C), one could see how Case (iv) struggled with the fitting—its RT PDFs were skewed leftward away from the data (Fig. 2C, bottom). If we consider time-variant gain on the DDM’s drift rate as increasing signal over time (within a trial), decisions made later within a trial are going to consist of more correct choices than errors. However, the monkey data generally has slower error RTs, which this model struggles to fit. This led to RT distributions, especially error RT distributions, not being properly fitted by Case (iv). This was observed in our previous work (Tan et al., 2023).

The model squared error and AIC for each case again showed that Cases (i) and (ii) provided the best fit, with Case (ii) perhaps gaining a slight advantage, whereas Cases (iii) and (iv) performed significantly worse (*p* < 0.05). Next, we compared the normalised fitted model parameters for all cases. We found that the parameter ${S}_{{\mathrm{y}}}$ for Case (iv) attained too high a value as compared to those of the other cases while ${S}_{{\mathrm{x}}}$ reached near the fitting boundary (i.e. 0) (Fig. 2E). As the parameter ${S}_{{\mathrm{y}}}$ controls the amplitude of the gain function (Eq. (3)), one could see that for small ${S}_{{\mathrm{x}}}$, the gain continued to rise linearly over a long period of time (Fig. 2E, inset).

Thus, the above results support our previous simulated results for the monkey data (Tan et al., 2023). Specifically, at least for the monkey data, time-variant gain only on drift rate enhances the signal at later times, leading to more correct but slower RTs, while time-variant gain only on noise term (with or without dependency on signal/stimulus $c$) leads to opposite model correct-vs-error RT trend (Fig. 1A). We shall next further investigate the two other species.

### Minimal and Indistinguishable Time-Variant Gain Effects for Human Data

**Fig. 3:**
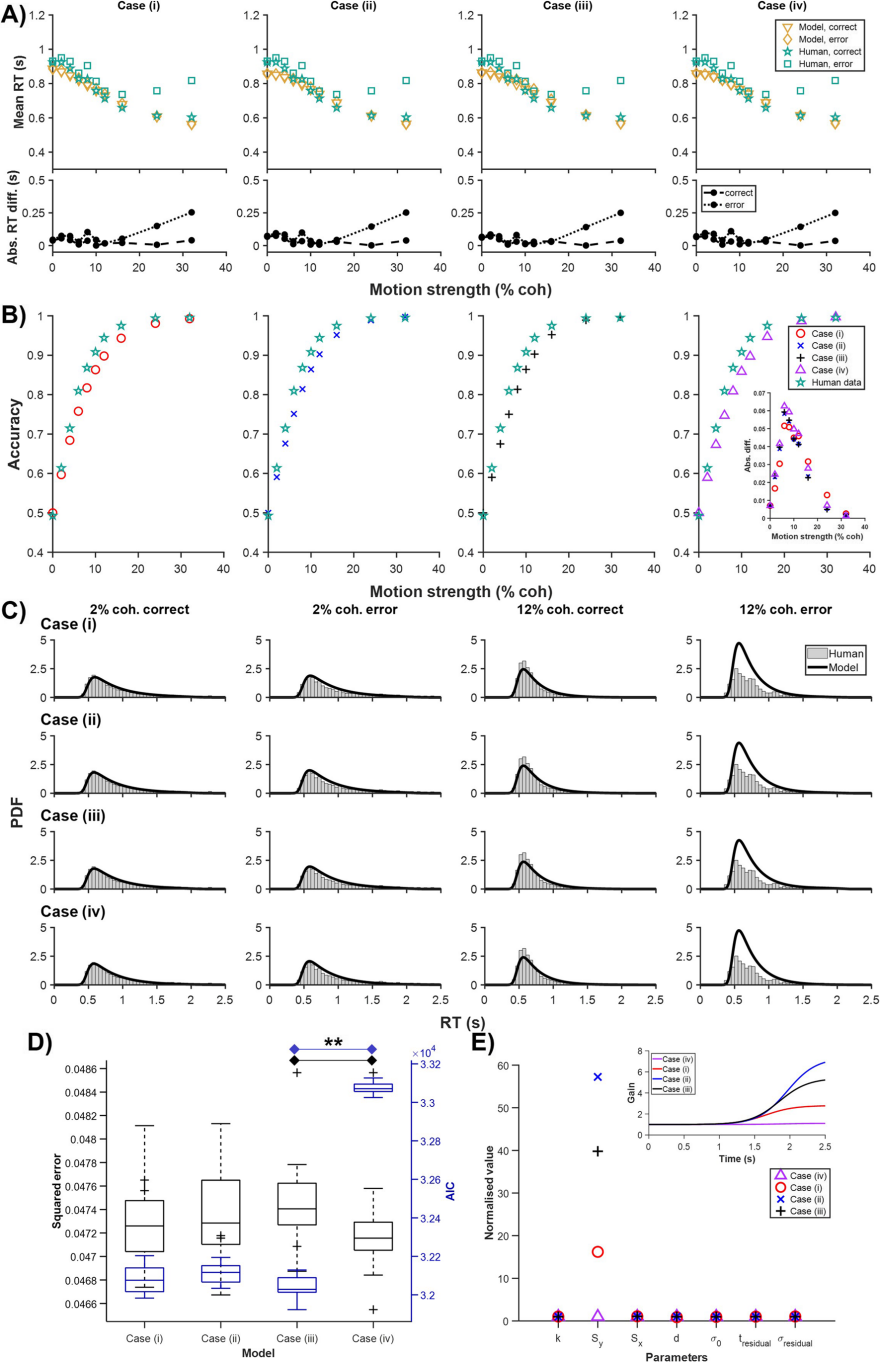
Minimal and indistinguishable time-variant gain effects for human data. Estimated models for four cases based on human choice behavioural data (choice accuracy and reaction time distributions) in Shevinsky and Reinagel (2019). Labels as in Fig. 2. **p < 0.01 in D

In the human data of Shevinsky and Reinagel (2019), error RTs were generally slower than correct choices, as in the monkey (Roitman & Shadlen, 2002) data, albeit only slightly (Fig. 3A). Interestingly, for very high motion strengths, human error RTs increased with increasing motion strengths, unlike in the monkey data. This could be due to high inter-participant variability.

Figure 3A shows the trend of the mean correct and error RTs across different motion strengths for all four cases of the model. One could observe that none of the cases provides good RT fit (Fig. 3A). All cases struggled with exhibiting slower error RTs, specifically for higher motion strengths. In terms of choice accuracy, all cases performed more poorly at intermediate motion strengths (Fig. 3B). The RT PDFs showed that the RT PDFs for the higher motion strength (12% coherence) were not captured well by all the cases (Fig. 3C).

The model squared errors were very close to each other, with Case (iv) providing the best fit only marginally (Fig. 3D). However, the AIC analysis revealed a different aspect: even though Case (iv) had the lowest squared error, it exhibited a significantly higher AIC value of 33,075 (*p* < 0.001), compared to the other cases (Cases (i) to (iii)), which hovered around 32,100, without any notable differences. When we compared the normalised fitted model parameters for all cases, we found that the amplitude parameter ${S}_{{\mathrm{y}}}$ differed the most across all the cases (Fig. 3E), and this was further evidenced in the fitted time-variant gain function timecourse (Fig. 3E, inset). Interestingly, the time-variant gain for Case (iv) was the smallest, very close to the value of 1, i.e. almost minimal gain influence. This was not surprising, given that the majority of error RTs were generally rather close to the correct RTs in the human data (except for high motion strengths, which had substantially fewer error trials).

### Time-Variant Gain on Drift Rate Accounts for Rat Data Best

**Fig. 4:**
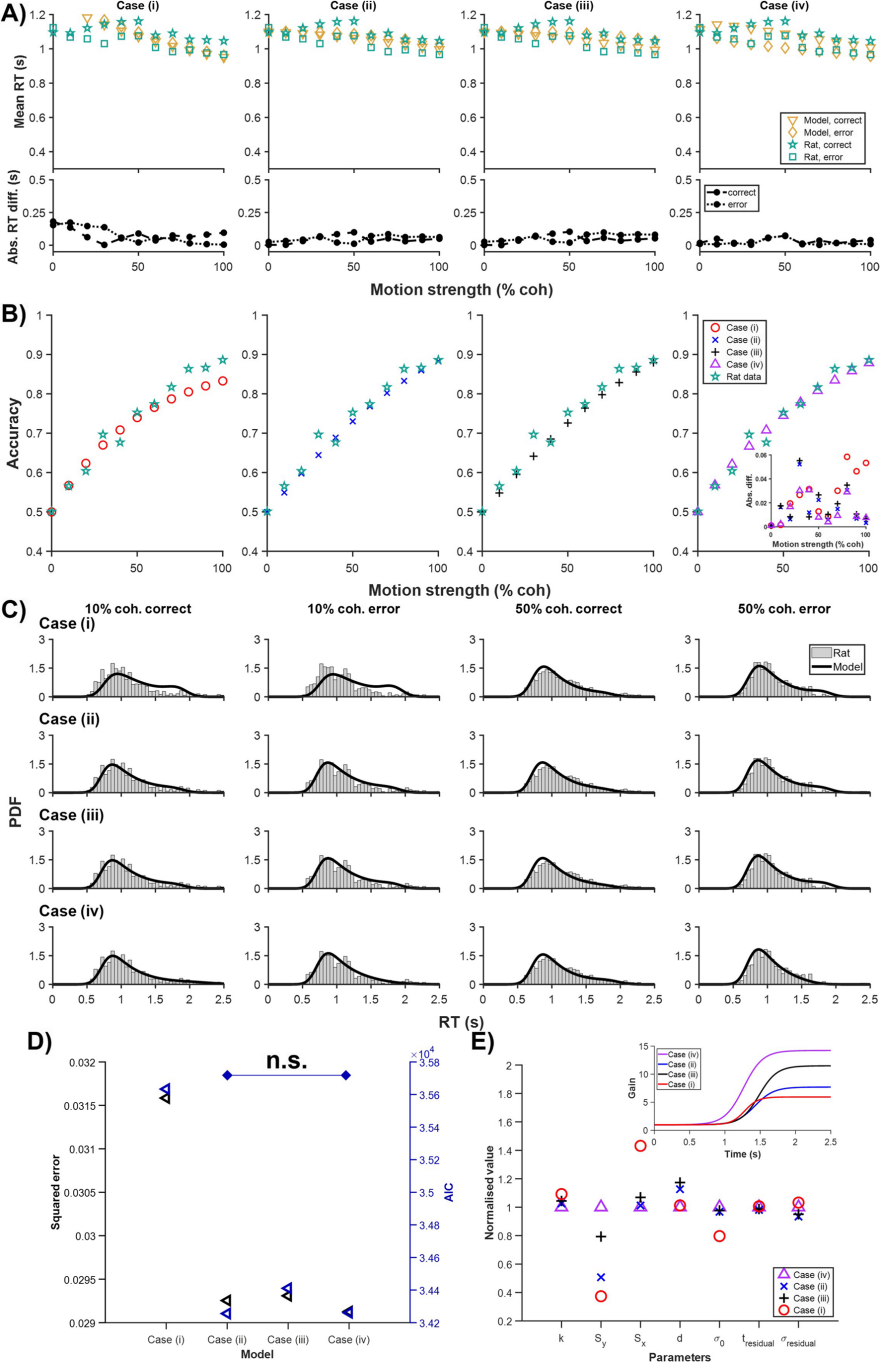
Time-variant gain on drift rate fits rat data best. Estimated models for four cases based on rat choice behavioural data (choice accuracy and reaction time distributions) in Shevinsky and Reinagel (2019). Labels as in Fig. 2

In the rodent data of Shevinsky and Reinagel (2019), error RTs were generally faster than correct RTs as motion strengths increased, unlike the above primate data (Fig. 4A). One could observe that Case (iv) provided the best fit for correct RTs, error RTs, and choice accuracy (Fig. 4A–C). Further, Cases (i)–(iii) were unable to separate correct and error RTs, while Case (i) was unable to fit choice accuracy for high motion strengths.

The model squared error again showed that Case (iv) provided the best fit while Case (i) had the worst fit (*p* < 0.05) (Fig. 4D). Complementing this, the AIC analysis corroborated Case (iv)’s superior fitting, as it, alongside Case (ii), exhibited the lowest mean AIC values at around 34,260, significantly lower than those of Cases (i) and (iii) (*p* < 0.05). As in the other datasets, the normalised fitted model parameters for all cases revealed the amplitude parameter ${S}_{{\mathrm{y}}}$ differed the most across all the cases (Fig. 4E), and this was further supported in the fitted time-variant gain function timecourse (Fig. 4E, inset). Moreover, Case (i) showed substantial deviation of values for model parameters ${S}_{{\mathrm{x}}}$ (slope of gain function) and ${\sigma }_{0}$ (noise level). With Case (iv), we have provided an alternative cognitive model (time-variant gain on drift rate) for the rat data in Shevinsky and Reinagel (2019).

## Discussion

In this work, with a focus on introducing an alternative parsimonious model, we investigated whether time-variant gain only on the noise term of a DDM leads to slower error RTs than correct RTs, while time-variant gain only on the drift rate of a DDM generates faster errors (Fig. 1A). Importantly, our approach focused on identifying general trends and effects consistent across subjects in each species. This perspective was crucial for highlighting the utility of group-level modelling in revealing broad behavioural trends in decision-making processes. Intuitively, one can consider time-variant gain on the DDM’s noise term as increasing the noise over time (within a trial), causing some form of signal-independent urgency to force a choice. Thus, decisions made at a later time (within a trial) are going to consist of more errors than correct ones, hence slower decisions (Fig. 1A). The effect is similar to the collapsing decision bound models (Ditterich, 2006; Drugowitsch et al., 2012; Hawkins et al., 2015).

In contrast, time-variant gain on the drift rate or signal effectively increases the signal strength over time, leading to improved accurate choices but at later times, as compared to error choices with the same stimulus or signal (Fig. 1A). One may be tempted to consider time-variant gain only on drift rate as a form of temporal enhancement of sensory signals, e.g. via attentional or arousal mechanisms (e.g. Smith & Ratcliff, 2009). However, one cannot associate its equivalence to collapsing bound DDM (Ditterich, 2006; Drugowitsch et al., 2012; Hawkins et al., 2015) despite their intuitive similarity. This is due to the choice biases (towards correct decisions) already inbuilt within the drift rate.

In this study, these separate time-variant gain mechanisms, together with the original time-variant gain DDM (model 4) of Ditterich (2006), were evaluated on monkey, human, and rat choice behavioural data performing the same motion discrimination task. Model parameter optimisation was conducted separately on the datasets. For monkey behavioural data from Roitman and Shadlen (2002), we found DDM with time-variant gain on both drift rate and noise term (Cases (i) and (ii)) to fit the data the best (Fig. 2). Interestingly, with regard to behavioural data, we found that even when noise term was independent of motion strength, the model could still capture the data almost equally well as the model (4) in Ditterich (2006), if not slightly better (Fig. 2D). We also found DDM with time-variant gain only on noise term (Case (iii)) could exhibit slower error RTs even though the fit was not as good, while time-variant on drift rate was unable to show that (Fig. 2A). This supported our earlier computational simulation observations (Tan et al., 2023).

For human behavioural data, our results showed that DDM with time-variant gain only on drift rate (Case (iv)) gave the best fit (Fig. 3). However, the fitting fitness (squared error) was comparable across all the cases (Fig. 3D). Interestingly, the AIC analysis revealed that despite Case (iv) having the lowest squared error, it exhibited a notably higher AIC, indicating a potential overfitting compared to Cases (i), (ii), and (iii), which had AIC values without significant differences. This suggests that while Case (iv) might provide a marginally better fit in terms of squared error, its complexity does not necessarily translate into a proportionally better model for the data. Further, the best-fitted case had minimal gain influence (with values near 1) (Fig. 3E). This lack of distinguishability across the cases and the minimal gain effects (of the best-fitted case) could be due to the relatively smaller difference between correct and error RTs, possibly caused by high variability among the human participants. Other human studies (e.g. Palmer et al., 2005; Smith & Ratcliff, 2022) also showed small differences in correct and error RTs, and that a standard DDM suffices to account for human choice behaviour. In other modelling studies such as in Nguyen and Reinagel (2022), response bias or across-trial variability in the drift rate is an alternative approach to account for the finer aspects of human behavioural data.

With regard to rat behavioural data, we found the model with time-variant gain only on drift rate provided the best fit (Fig. 4), consistent with our earlier observation on generating faster error RTs (Tan et al., 2023). This assertion was further strengthened by the AIC analysis, which indicated that Cases (ii) and (iv), particularly Case (iv), had the most favourable balance between model complexity and fit, as evidenced by their significantly lower AIC values compared to Cases (i) and (iii) (*p* < 0.05). Interestingly, models with time-variant gain on both drift rate and noise (Cases (i) and (ii), especially Case (i)) were unable to replicate the data as well. This could perhaps be due to the equal modulatory factors on these two terms in the models, hence permitting less flexibility.

By comparing the best-fitted case for each species, we found substantial variability in the amplitudes of the time-variant gain functions (Fig. S1). Specifically, the time-variant gain amplitude decreases from rats, to monkeys and then humans. An interesting side observation was that the gain function for monkeys changed substantially only within a relatively short duration of time within a critical decision formation epoch. This duration was comparable to the modelled gain time constant (~ 190 ms) in Niyogi and Wong-Lin (2013), which used a more biologically based decision model to account for the data of Roitman and Shadlen (2002). Taken together, our results shed light on the possible different mechanisms or strategies during perceptual decision formation across the species.

Our work has focused on capturing choice behaviour in different species, but the neural network mechanism(s) to separately instantiate the gain modulation of either the network’s signal or noise is still unclear, especially in more biologically based decision models (e.g. Wang, 2002; Wong & Wang, 2006) wherein signal and noise reside within neuronal input–output functions. In particular, in more biological models of decision-making (e.g. Wong and Wang (2006)), the neural response (e.g. neural firing rate), $f$ is dependent on the input–output or transfer function $F$, generally some nonlinear function, such that $f=F({I}_{{\mathrm{total}}})$, where ${I}_{{\mathrm{total}}}$ is the total input (current) to the neurons. Time-variant gain, $\gamma (t)$, typically operates such that $f=\gamma \left(t\right) F({I}_{{\mathrm{total}}})$, i.e. acting as a multiplicative gain factor on the input–output function (e.g. Niyogi and Wong-Lin (2013)).

Although $F$ is generally nonlinear, the operating regime may span around the approximately linear part of the function (e.g. see Wong and Wang (2006)). For clarity of explanation, suppose this is the case, and that the noise is additive and separate from the signal, such that ${I}_{{\mathrm{total}}}={I}_{{\mathrm{signal}}}+{I}_{{\mathrm{noise}}}$, then $f=\gamma \left(t\right) F\left({I}_{{\mathrm{total}}}\right)={\gamma \left(t\right) I}_{{\mathrm{signal}}}+\gamma \left(t\right) {I}_{{\mathrm{noise}}}$, thus demonstrating the apparent difficulty in separate gains on the input signal and noise terms.

As mentioned above, noise in decision systems can be contributed by internal noise (in the brain) or external (stimulus) noise, or both, i.e. ${I}_{{\mathrm{noise}}}={I}_{\mathrm{internal \: noise}}+{I}_{\mathrm{external \: noise}}$. If there is a time-variant gain acting on the sensory evidence signal, which may constitute the major source of noise (as possibly in non-human primates), then both decision signal (drift rate) and noise may be affected by this time-variant gain. In contrast, if there is a different internally generated major source of noise (as possibly in rats), then time-variant gain may effectively not operate much on the noise. This still leaves us with the question on how time-variant gain only on drift rate may possibly arise.

From several neurophysiological studies, it is known that noise in neurons can be attributed to the barrage of balanced excitatory and inhibitory (*E*/*I* balance) synaptic currents such that the average synaptic input can be relatively small while its variance (noise) is large (see e.g. Okun and Lampl (2009), and also references in Niyogi and Wong-Lin (2013)). Transient *E*/*I* imbalance, e.g. due to unequal (heterogeneous) chemical neuromodulation (e.g. Eckhoff et al., 2009; McBurney-Lin et al., 2019), may lead to larger proportional change in the average signal but smaller proportional change in its variance (noise). This may effectively lead to time-variant gain on the DDM’s drift rate being affected more than on its noise. For example, transient activity of the neuromodulator norepinephrine has been known to be associated with enhanced neural signal-to-noise ratio in perceptual tasks (Aston-Jones & Cohen, 2005).

In this work, we have focused on group-level analyses as our main aim was to explore how introducing time-variant gain parameters could impact the fit of decision-making models to empirical data, rather than seeking a generalised model to apply to new datasets. Future work can apply individual-level modelling rather than group level to investigate the interplay of individual differences and varied strategies in decision-making. Additionally, further research could explore larger datasets, enhancing model robustness and validation. Implementing cross-validation methods would provide deeper insights into the generalisability of decision-making models. These directions, vital for advancing our understanding, promise to address current limitations and broaden our comprehension of diverse choice behavioural dynamics. Taken together, from a cognitive computational science viewpoint, we have shown that time-variant gain on the drift and noise term for a DDM can differently affect choice behaviour, account for different species’ choice behaviour, and can be useful for systematic fitting of choice behavioural data.

## Supplementary Information

Below is the link to the electronic supplementary material.

Supplementary file1 (DOCX 33.5 KB)
