# Supplementary material for: Of Rodents and Primates: Time-Variant Gain in Drift–Diffusion Decision Models
Source: Comput Brain Behav. 2024 Jan 11;7(2):195–206. doi: 10.1007/s42113-023-00194-1 (PMC11111503; doi:10.1007/s42113-023-00194-1)
Supplement: Supplementary file 1 — Supplementary file1 (DOCX 33.5 KB) [file 42113_2023_194_MOESM1_ESM.docx]

**Of rodents and primates: Time-variant gain in drift-diffusion decision models**

**Supplementary Information**

Abdoreza Asadpour, Hui Tan, Brendan Lenfesty, KongFatt Wong-Lin

# Supplementary Note 1: Best-fitted time-variant gain characteristics vary widely across species

After separately fitting the model to different species and cases, we compared the identified gain functions from the best-fitted case for each species: Case (ii) for monkeys, Case (iv) for humans, and Case (iv) for rats. We should bear in mind that although the experimental task paradigm used was similar for the species, the detailed conditions might differ. Nevertheless, it is still worth comparing the best-fitted model, which may in turn provide insights into the underlying time-variant gain mechanisms.

The first to be noticed was that the gain function of the best-fitted case for rats had a much higher amplitude than that of the other two species (Fig. S1A). The gain function of the best-fitted case for monkeys started and saturated earlier than that of the rats such that the increasing gain effect occurred only within a relatively short duration (for ~0.5 s). For humans, the effects of time-variant gain were almost negligible. These observations were also supported at the level of specific best-fitted gain parameters, in which the amplitude parameter $S_{y}$ and the time-shift parameter $d$ were relatively more varied across the three species (Fig. S1B, Table S1).

**Fig. S1 Variability in gain function and parameters of best-fitted cases across species.** (A-B) Timecourse (A) and normalised parameter values to monkey Case (ii) (B) of time-variant gain function of best-fitted case for each species.

**Table S1 Parameter values of best-fitted cases for the species.** Bold: outstanding values in the context of each parameter, highlighting the most notable results within each species.

|  | $\boldsymbol{k}$ | $\boldsymbol{S}_{\boldsymbol{y}}$ | $\boldsymbol{S}_{\boldsymbol{x}}$ | $\boldsymbol{d}$ | $\boldsymbol{\sigma}_{\boldsymbol{0}}$ | ${\bar{\boldsymbol{t}}}_{\boldsymbol{residual}}$ | $\boldsymbol{\sigma}_{\boldsymbol{residual}}$ |
| --- | --- | --- | --- | --- | --- | --- | --- |
| **Monkey, Case (ii)** | 12.472 | 2.021 | 9.429 | **0.511** | 0.870 | 0.257 | 0.088 |
| **Human, Case (iv)** | 20.227 | 0.110 | 4.609 | **1.878** | 1.498 | 0.403 | 0.035 |
| **Rat, Case (iv)** | 2.032 | **13.220** | 7.394 | **1.264** | 1.468 | 0.640 | 0.104 |
